# pH-Sensitive Starch-Based Hydrogels: Synthesis and Effect of Molecular Components on Drug Release Behavior

**DOI:** 10.3390/polym12091974

**Published:** 2020-08-31

**Authors:** Juan Carlos Quintanilla de Stéfano, Vanessa Abundis-Correa, Sergio Daniel Herrera-Flores, Alejandro J. Alvarez

**Affiliations:** School of Engineering and Sciences, Tecnologico de Monterrey, Av. Eugenio Garza Sada 2501, Monterrey 64849, NL, Mexico; A01139081@itesm.mx (J.C.Q.d.S.); A01280646@itesm.mx (V.A.-C.); A01280845@itesm.mx (S.D.H.-F.)

**Keywords:** hydrogel, controlled drug release, starch copolymers, caffeine, swelling behavior

## Abstract

The drug release behavior of pH-sensitive starch-based hydrogels was systematically studied. Hydrogels were synthesized by copolymerization of acrylic acid (AA) and other acrylate comonomers onto the starch backbone. The hydrophilic agents 2-hydroxy ethyl methacrylate (HEMA), and acrylamide (AAm), as well as the hydrophobic butyl-methacrylate (BMA), were utilized as comonomers. Methylene-bisacrylamide (MBA) was employed as a crosslinking agent. The synthesized hydrogels were loaded with caffeine as a model drug. The effects of the hydrophobic/hydrophilic character of the comonomers and chemical crosslinking on the swelling capacity and the release rate of caffeine were investigated. The use of the crosslinking agent and hydrophobic monomers decreased the swelling capacity of the hydrogels. The release rate of caffeine increased with the presence of a hydrophobic monomer. The fastest release was obtained with the AA/BMA/AAm formulation, and the slowest release was observed with the AA/HEMA/AAm formulation. The transport mechanism was controlled by Fickian diffusion in formulations containing AAm, and controlled by the polymer-relaxation mechanism in formulations containing MBA. Overall, our results showed that the swelling and drug delivery behavior can be tuned by varying the chemical composition of the copolymer formulations. These starch-based hydrogels can be useful as drug delivery devices in many biomedical applications.

## 1. Introduction

Hydrogels are hydrophilic, three-dimensional polymer networks with a high water absorption capacity [1]. They have received considerable attention due to their biomedical applications such as drug delivery and tissue engineering [2,3]. Smart hydrogels are responsive to various physical stimuli, including temperature, pH, light, and ionic strength [4,5,6,7,8]. Lately, smart hydrogels made from natural polymers have attracted increasing interest from researchers due to their biocompatibility and biodegradability [9]. The use of polysaccharides such as starch [10,11,12,13], cellulose [14,15], and chitosan [16] has been recently reported. Starch-based hydrogels can be synthesized by free-radical polymerization [17,18]. The copolymerization of specific monomers onto starch is an attractive and effective method to synthesize stimuli-responsive natural-based hydrogels [19,20,21,22].

In the controlled delivery systems based on hydrogels, the release of the drug occurs by the diffusion of the drug through the polymer. The body fluids (primarily water) flow inside the polymer, dissolve the drug, and diffuse back through the swollen hydrogel matrix. The rate of diffusion of water into the polymer controls the rate of release of the drug. Some of the factors that determine the rate of diffusion are the nature of the comonomers present on the copolymer and the structure of the matrix [23]. In particular, it depends on the polarity of the polymer segments, the crosslinking density, and the presence of bulky comonomer groups (stereochemical structure). Those factors also control the amount of water that can be absorbed inside the hydrogel matrix [24,25].

The development of stimuli-sensitive hydrogels for biomedical applications has gained attention in recent times. Amongst the stimuli-responsive hydrogels, pH-sensitive hydrogels are the most studied systems. Many research works have focused on the use of poly acrylic acid as a suitable candidate for pH-sensitive-based drug delivery systems. Many examples of drug delivery applications using acrylic acid copolymerized with other monomers in natural polymers have been published recently. Gupta et al. prepared pH-sensitive hydrogels for the stomach-targeted drug delivery of clarithromycin [26]. Acrylic acid was used to graft chitosan by free radical polymerization, blended with poly(vinyl pyrrolidone), and crosslinked with glutaraldehyde and *N*,*N*-methylenebisacrylamide. Sun et al. synthesized pH-sensitive hydrogels for the delivery of theophylline and acetylsalicylic to treat respiratory tract diseases [27]. Acrylic acid was grafted to hemicellulose and cross-linked with *N*,*N*-methylenebisacrylamide. Das et al. developed acrylic acid-grafted guar gum/beta-cyclodextrin-blended hydrogels crosslinked with tetraethyl orthosilicate for the intestine-targeted delivery of dexamethasone [28]. The hydrogel was intended for the treatment of inflammatory bowel disease and ulcerative colitis. Zhang et al. successfully synthesized pH-sensitive acrylic acid-based hydrogels for the safe oral delivery of insulin [29]. The pH-sensitive hydrogels were prepared by grafting acrylic acid onto the chains of carboxymethyl chitosan loaded with insulin. Ghaffar et al. prepared pH-sensitive acrylic acid-grafted starch hydrogels to study the controlled release of rutin in the colon [30]. Kim and Oh developed crosslinked glycidyl methacrylate dextran and poly(acrylic acid) pH-sensitive hydrogels for colon specific drug delivery [31]. The hydrogels were intended to treat colorectal cancer, Crohn’s disease, and ulcerative colitis.

The recent advances in drug delivery applications using acrylic acid demonstrate that pH-sensitive behavior can be engineered by grafting or copolymerizing certain monomers on the polymeric chains, and can be used for site-specific drug delivery to improve the bioavailability and efficacy ratio of existing drugs. A key role in the swelling and drug release behavior of the hydrogel is the interaction of the hydrophobic and hydrophilic moieties present in the polymeric matrix. Our work aims to contribute to a better understanding of the effect of the hydrophobic/hydrophilic character of the monomers on the hydrogel properties. The characterization of the release behavior of caffeine in starch-based hydrogels as a function of the hydrophobic/hydrophilic character of acrylic monomers has not been previously reported, and the contribution is relevant for the development of new stimuli-sensitive hydrogels.

Caffeine was used as a model drug in this study. Caffeine is one of the most consumed drugs worldwide, with large application in pharmaceuticals, food, and cosmetics. It is used as a stimulant of the central nervous system, in the prevention and cure of skin cancer, and as an anti-cellulite drug [3]. It has been reported that the regular consumption of caffeine can reduce the probability of suffering from liver disease [32]. However, due to its easy dissolution in aqueous media, rapid absorption, distribution, and elimination in the body, it requires frequent oral administration to maintain an adequate blood concentration. A drug delivery system is desirable for the controlled sustained release of caffeine. The use of hydrogels as platforms for the controlled release of caffeine is explored in this study.

**Figure 1:**
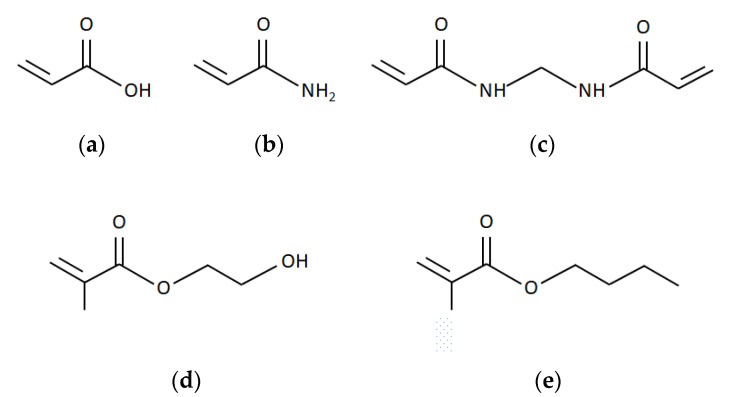
Chemical structure of the monomers and crosslinker: (**a**) Acrylic acid; (**b**) Acrylamide; (**c**) Methylene bisacrylamide; (**d**) 2-Hydroxy ethyl methacrylate; (**e**) Butyl methacrylate.

In the present work, different copolymers of acrylic acid (AA), 2-hydroxy ethyl methacrylate (HEMA), butyl methacrylate (BMA), acrylamide (AAm), and methylene bisacrylamide (MBA) in starch were prepared. The effects of the hydrophobic/hydrophilic character of the comonomers and chemical crosslinking on the swelling capacity and the release rate of caffeine were investigated. The chemical structures of the monomers are presented in Figure 1.

The monomers AA, AAm, and HEMA have hydrophilic carboxyl groups. AA and HEMA also have a polar hydroxyl group, with high hydrophilicity. The side-chain of the monomer BMA exhibits a hydrophobic character. MBA is used as a crosslinking agent. Their end vinyl groups may react synchronously with polymer chains during the chain preparation.

## 2. Materials and Methods

### 2.1. Materials and Chemicals

Starch from corn (≤15% loss on drying), acrylic acid (99%), 2-hydroxy ethyl methacrylate (97%), butyl methacrylate (99%), potassium persulfate (99%), methylene bisacrylamide (99%), and acrylamide (99%) were purchased from Sigma Aldrich (Saint Louis, MO, USA). Buffer solutions of pH 4 and pH 7 were obtained from Fisher Scientific.

### 2.2. Synthesis of Hydrogels

The copolymerization reaction was carried out in a 2-neck jacketed glass reactor at 80 °C. Starch (2 g) was added to distilled water (35 mL) and stirred for 30 min at 300 rpm. The procedure continued with the addition of the comonomers and crosslinker. The relative amount of AA, HEMA, BMA, AAm, and MBA utilized in each experiment is shown in Table 1. Afterwards, potassium persulfate (0.1 g) was added as an initiator. The reaction mixture was stirred for 60 min. The resulting hydrogel was cooled down to room temperature. The pH was adjusted to 8 through the addition of NaOH (1 N) solution. Then, the final product was poured in 200 mL of ethanol for 24 h. At the end, the hydrogel was vacuum filtered, dried in an oven at 70 °C for 24 h, and stored in a desiccator away from moisture and light. All experiments were triplicated.

### 2.3. FTIR Spectral Analysis

The starch-based hydrogels were characterized by a Fourier transform infrared spectrometer Perkin Elmer (Waltham, MA, USA) model Spectrum 400. Spectra were recorded in the wavenumber range of 4000–700 cm^−1^.

### 2.4. Swelling Capacity Measurements

Dry samples of the hydrogel with a weight of 0.05 g ± 0.001 g were placed in 10 mL of aqueous solutions with pH 2, 3, 4, 5, 6, 7, 8, 9, and 10. After 24 h, the samples were vacuum filtered and weighed. Equation (1) was used to determine the swelling capacity of the hydrogels, where $M_{o}$ is the mass of the dry hydrogel and M is the mass of the hydrogel after swelling.

(1) $$\mathrm{Swelling}\;\mathrm{capacity}\;(g/g)=\left(\frac{M}{M_{o}}-1\right)$$

Dilution of NaOH 0.1 N solution with distilled water was used to obtain aqueous solutions with a pH greater than 8, whereas dilution of HCl 0.1 N solution with distilled water was used to obtain aqueous solutions with a pH lower than 6.

### 2.5. Rheological Measurements

Rheological properties were measured using a rotational rheometer (Physica MCR 301, Anton Paar, Ostfildern, Germany) equipped with a cone-and-plate geometry (diameter of 25 mm). Experiments were conducted at 25 ± 0.1 °C. The storage modulus (*G*′) was obtained as a function of the angular frequency.

### 2.6. Drug Loading to the Polymer Matrix

Samples of 0.10 g ± 0.01 g of dry hydrogels were immersed in an aqueous solution of caffeine with a concentration of four grams per liter at 25 °C. After 72 h, the swollen hydrogels loaded with drug were vacuum filtered, placed in a vacuum oven, and dried under vacuum at 37 °C.

### 2.7. In Vitro Drug Release

The drug release experiments were carried out by transferring the previously loaded hydrogels into phosphate buffer solutions at 37 °C. All experiments were triplicated for each starch-based copolymer. Dry samples of the loaded hydrogel were immersed in 15 mL of buffer solution pH 4 and in 40 mL of buffer solution pH 7 at 37 °C. At specific time intervals, samples of the buffer solution were withdrawn. The concentration of the released drug in the buffer solution was determined by spectrophotometry [33] using a UV-Vis spectrophotometer (Model HACH DR 6000, Loveland, CO, USA). The caffeine release percentage was calculated using the following equation, where $M_{co}$ is the initial mass of caffeine loaded in the hydrogel, and $M_{ct}$ is the mass of caffeine released at time t:

(2) $$\mathrm{Caffeine}\;\mathrm{release}\;(\%)=\left(\frac{M_{ct}}{M_{co}}-1\right)*100$$

The caffeine release was measured after a fixed interval of time, and the release dynamics of the model drug were studied. The caffeine cumulative release data was fitted to the Ritger−Peppas model [34].

## 3. Results and Discussion

### 3.1. Hydrogel Synthesis

**Figure 2:**
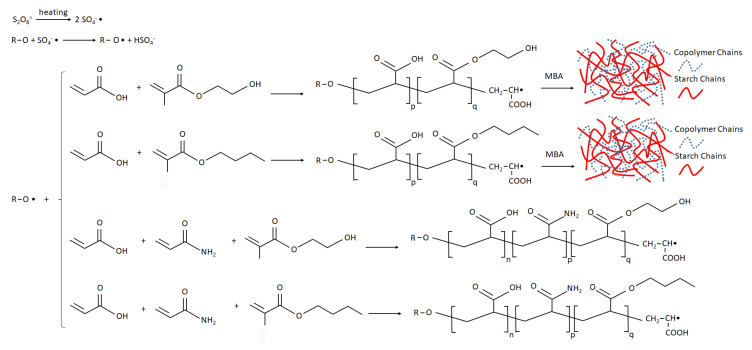
Mechanism of copolymerization of HEMA/AA/MBA, BMA/AA/MBA, HEMA/AA/AAm, and BMA/AA/AAm on starch backbone.

The composite hydrogels were prepared by copolymerization of acrylate comonomers onto starch. Potassium persulfate was used as a free radical initiator. The persulfate initiator is decomposed at 80 °C to generate sulfate anion radicals. The radicals remove hydrogen from the hydroxyl group of starch to form alkoxy radicals. The monomer molecules (AA, and HEMA or BMA) that surround the reaction sites become acceptors of starch radicals, resulting in chain initiation. Then, they become free radical donors to other molecules during the chain propagation. When the crosslinking agent MBA is present in the system, the end vinyl groups of MBA react with polymer chains, forming a three-dimensional crosslinking network. If AAm is present in the system (instead of MBA), the formed hydrogel is a copolymer without the chemically crosslinked structure. The mechanism of copolymerization is outlined in Figure 2 [14].

### 3.2. FTIR Analysis

**Figure 3:**
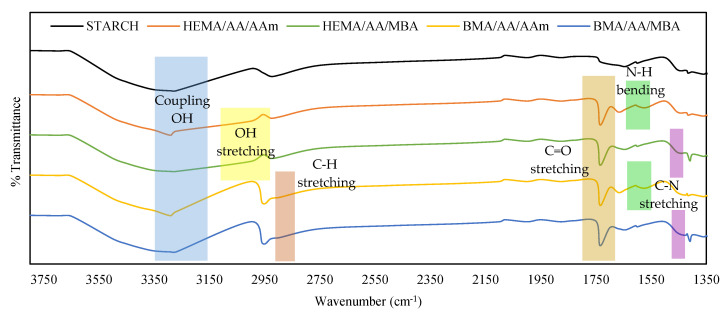
FTIR spectra of starch, HEMA/AA/AAm, HEMA/AA/MBA, BMA/AA/AAm, and BMA/AA/MBA hydrogels.

The mixture of monomers was copolymerized onto starch backbones in a homogeneous medium using free-radical polymerization. The reaction was confirmed by comparing the FTIR spectra of the starch substrate with that of the copolymerization products (see Figure 3).

In the spectra of all hydrogels, the characteristic band at 1735 cm^−1^ is associated with C=O stretching vibrations of the acrylic acid units. This characteristic band is not present in the starch spectrum. The characteristic peak at 2958 cm^−1^ is due to the C–H stretching of the BMA units, and it is present in BMA/AA/AAm and BMA/AA/MBA hydrogels. The broad band between 3000 and 3100 cm^−1^ is attributed to the overlapping vibration of the OH stretching of HEMA, and it is present in the HEMA/AA/AAm and HEMA/AA/MBA hydrogels. The characteristic peak at 1568 cm^−1^ is attributed to the N–H bending in the AAm units, and it is present in HEMA/AA/AAm and BMA/AA/AAm hydrogels. Finally, the C–N stretching band at 1409 cm^−1^ is attributed to the MBA, and it is present in the HEMA/AA/MBA and BMA/AA/MBA hydrogels.

### 3.3. Swelling Characterization of Hydrogels

The ability to swell is a favorable property of polymer hydrogels. Its characterization has a great relevance, mainly due to the impact of the swelling behavior on the surface properties, mobility, solute transport mechanism, and mechanical properties of the hydrogel.

#### 3.3.1. Effect of the pH on the Swelling Behavior

**Figure 4:**
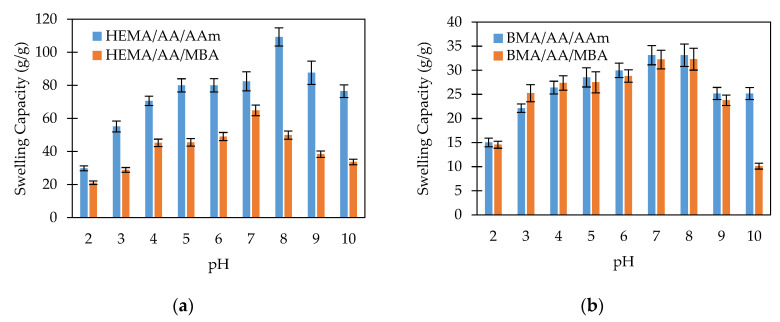
Swelling capacity of the hydrogels as a function of the pH. Effect of crosslinking: (**a**) Comparison of HEMA/AA/AAm and HEMA/AA/MBA hydrogels; (**b**) Comparison of BMA/AA/AAm and BMA/AA/MBA hydrogels.

**Figure 5:**
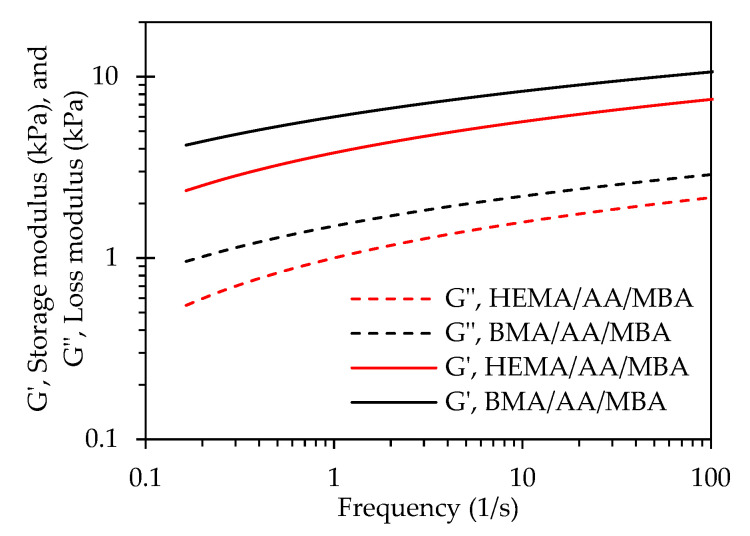
Storage modulus (*G*′) and loss modulus (*G*″) of the hydrogels as a function of the angular frequency.

In order to investigate the sensitivity of the starch-based hydrogels to pH, the swelling capacity of the hydrogel was studied at various pHs ranging from 2 to 10. The swelling behavior studies indicated that all hydrogel formulations were sensitive to the pH of the liquid media. The swelling capacity reached a maximum value close to the neutral or physiological pH. The swelling values decreased as the pH moved to lower (2 < pH < 7) or higher (8 > pH > 10) values. The starch-based hydrogels contain anionic-type groups such as carboxylate and, in some cases, carboxamide groups. Under acidic solutions (pH < 6), most of those anions are protonated. The hydrogen bonding interaction among the anionic groups is strengthened, and the hydrogel network tends to shrink. Consequently, the swelling capacity values are decreased. On the other hand, at neutral pH values, some of the anionic groups are ionized. Thus, the hydrogen bonding interactions are broken, and the electrostatic repulsion between the anionic groups is increased. As a result, the polymer network tends to swell, and the swelling capacity is high. Finally, at higher pH values (pH > 9), a charge screening effect occurs due to the excess of sodium ions in the liquid media. This excess shields the anion, prevents effective electrostatic repulsion, and causes a decrease of the hydrogel swelling. Similar swelling-pH behaviors have been reported in other hydrogel systems [15,16,35,36]. The swelling-pH behavior of the starch-based hydrogels is observed in Figure 4 and Figure 5.

#### 3.3.2. Effect of the Crosslinking on the Swelling Behavior

Figure 4 shows the swelling capacity of chemically crosslinked and non-chemically crosslinked hydrogels. In Figure 4a, the swelling behavior of HEMA/AA/AAm and HEMA/AA/MBA hydrogels is compared. Both hydrogels contain the hydrophilic monomers HEMA and AA, but HEMA/AA/MBA is chemically crosslinked. The swelling capacity of the chemically crosslinked hydrogel is significantly lower than that of the nonchemically crosslinked copolymer. For instance, the swelling capacity at pH 2 is 1.5 times greater in the HEMA/AA/AAm than in the HEMA/AA/MBA hydrogel, and the difference is even larger at physiological pH values. This fact can be attributed to the crosslinking density being higher in the chemically crosslinked hydrogel. Due to the chemical structure of MBA, the chemically crosslinked network of the HEMA/AA/MBA hydrogel is denser than HEMA/AA/AAm. The description of the swelling of the hydrogels at equilibrium is based on the minimization of the Gibbs free energy of the gel. Based on the Flory–Rehner theory, the minimum Gibbs free energy is reached when the elasticity and osmotic forces are balanced. Increasing the crosslinking density of the hydrogel leads to shorter chains between crosslinks. The elasticity force of the shorter chains during swelling develops faster than in the case of longer chains, and therefore the elasticity forces of the hydrogel become equal to the osmotic forces at a lower relative swelling. Consequently, the hydrogels with a higher crosslinking density exhibit a lower swelling capacity.

The rheological properties of the chemically crosslinked starch-based hydrogels are shown in Figure 5. The storage modulus is an indication of the ability of the hydrogel to store deformation energy in an elastic manner. As can be observed in Figure 5, the storage modulus (*G*′) of the BMA/AA/MBA gel is greater than for the HEMA/AA/MBA gel. The crosslinking density is defined as the number of chain segments between crosslinks per unit volume. The crosslinking density (*V_e_*) of the hydrogels was calculated using the values of the storage modulus (*G*′) determined from the rheological measurements. According to the theory of rubber elasticity [37], the crosslinking density of the hydrogels is directly proportional to the storage modulus (*G*′). Equation (3) was used to calculate the crosslinking density of the chemically crosslinked hydrogels [38].

(3) $$G^{\prime }=V_{e}\;\rho \;RT\;{\left(\nu_{2,r}\right)}^{2/3}{\left(\nu_{2,s}\right)}^{1/3}$$

where *ρ* is the polymer density, *R* is the universal gas constant, *T* is the absolute experimental temperature, *G*′ is the average value of the storage (elastic) modulus, *ν*_2,*r*_ is the polymer volume fraction after crosslinking, and *ν*_2,*s*_ is the polymer volume fraction of the crosslinked hydrogel in a swollen state.

The calculated values for the crosslinking densities of BMA/AA/MBA and HEMA/AA/MBA were 13.14 mol/m^3^ and 11.1 mol/m^3^, respectively. The higher storage modulus of BMA/AA/MBA when compared to HEMA/AA/MBA can be explained by the greater crosslinking density of the former system. Moreover, the hydrophilic interactions of the BMA moiety in the BMA/AA/MBA system are relatively stronger than the hydrogen bond interactions of the HEMA monomers in the HEMA/AA/MBA hydrogel, contributing to the greater mechanical strength of BMA/AA/MBA. Additionally, the higher swelling capacity of the HEMA/AA/MBA system when compared to BMA/AA/MBA can be related to the rheology data. The lower storage modulus of HEMA/AA/MBA can be associated to its lower crosslinking density and to the presence of the hydrophilic HEMA monomer, whose hydrogen bond interactions are relatively weaker than the hydrophobic interactions of the BMA moiety in the BMA/AA/MBA gel. The hydrophilic HEMA monomer leads to an increasing degree of swelling.

In contrast, Figure 4b shows the swelling behavior of BMA/AA/AAm and BMA/AA/MBA hydrogels. Both copolymers contain the hydrophobic monomer BMA, but BMA/AA/MBA is chemically crosslinked. In both hydrogels, the swelling capacity as a function of pH does not exhibit a pronounced change. For instance, the swelling capacity of BMA/AA/AAm at pH 2 is 1.04 times greater than that for the BMA/AA/MBA hydrogel at pH 2 and 1.03 times greater at pH 7. The lack of a response to the pH stimulus may be attributed to the hydrophobic attraction among the hydrophobic BMA segments that dominate the swelling behavior and decrease the ability of the hydrogel to swell.

#### 3.3.3. Effect of the Hydrophilic/Hydrophobic Monomers on the Swelling Behavior

**Figure 6:**
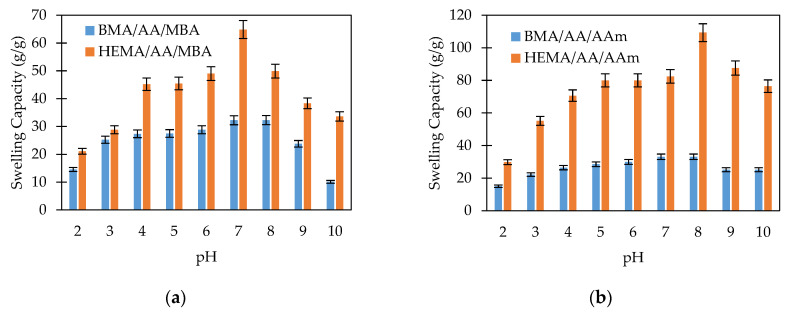
Swelling capacity of the hydrogels as a function of the pH. Effect of the hydrophilic/hydrophobic monomers: (**a**) Comparison of BMA/AA/MBA and HEMA/AA/MBA hydrogels; (**b**) Comparison of BMA/AA/AAm and HEMA/AA/AAm hydrogels.

The effect of the hydrophilic/hydrophobic character of the monomers on the swelling behavior of the copolymers was evaluated by comparing the swelling capacity of hydrogels with and without the hydrophobic BMA monomer. In Figure 6, it can be observed that the hydrogels synthesized with HEMA as the comonomer (HEMA/AA/AAm and HEMA/AA/MBA) exhibited a greater swelling capacity when compared to those produced with BMA as the comonomer (BMA/AA/AAm and BMA/AA/MBA). This behavior is explained by the chemical structure of both monomers. HEMA has –OH groups, which makes it hydrophilic and gives the copolymer the ability to create hydrogen bonds with water molecules. In contrast, the non polar BMA units inhibit the interaction with water, restrain the expansion of the hydrogel networks, and consequently decrease the water uptake.

### 3.4. Drug Release Behavior

**Figure 7:**
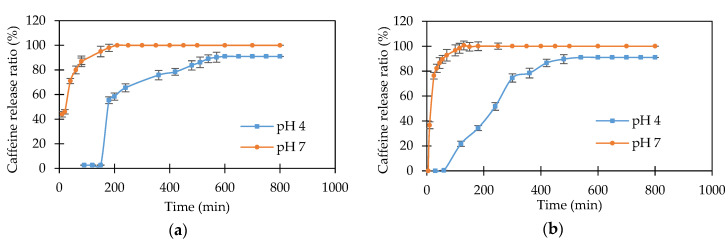
Drug release for caffeine into pH 4 and pH 7 buffer solution: (**a**) HEMA/AA/AAm; (**b**) HEMA/AA/MBA.

**Figure 8:**
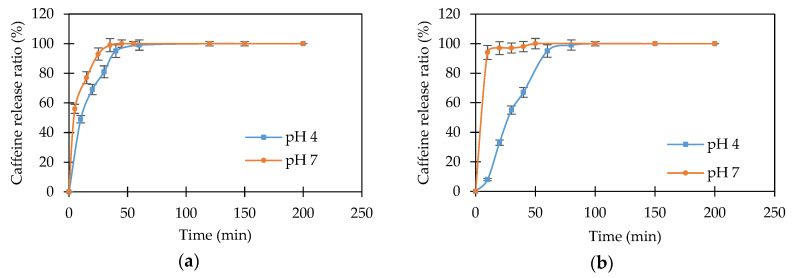
Drug release for caffeine into pH 4 and pH 7 buffer solution: (**a**) BMA/AA/AAm; (**b**) BMA/AA/MBA.

The release profile of caffeine molecules from starch-based copolymer (HEMA/AA/AAm, HEMA/AA/MBA, BMA/AA/AAm, and BMA/AA/MBA) hydrogels into pH 4 and pH 7 buffer solutions can be observed in Figure 7 and Figure 8. In these figures, it is readily apparent that the caffeine molecule has a much faster release at pH 7 than at pH 4. Figure 7a,b shows that caffeine release starts immediately after the hydrogels are immersed into pH 7 buffer and that it is completely released after 210 min and 130 min for the HEMA/AA/AAm and HEMA/AA/MBA hydrogels, respectively. However, the same hydrogels show a delay of 150 min and 60 min before the caffeine starts to be released in pH 4 buffer. After 210 and 130 min, approximately 58% and 25% of the preloaded caffeine is released from the HEMA/AA/AAm and HEMA/AA/MBA hydrogels, respectively.

This difference can be explained by the stimuli-response character of the hydrogels. At acidic pH values, the anionic groups on the starch-based hydrogels become protonated, thus eliminating the negative charge on these functional groups. The hydrogen bonding interaction among the anionic groups is strengthened, and the hydrogel network tends to shrink. Consequently, the swelling ability of the hydrogel is decreased and decreases the pore size in the matrix structure. Therefore, the release of caffeine proceeds at a slow rate inside the pH 4 buffer solution, and it takes up to approximately 600 min to complete the release in the HEMA/AA/AAm hydrogel. In contrast, at physiological pH values, the anionic groups on the hydrogel are ionized, increasing the electrostatic repulsion between them. Consequently, the polymer network swells, increasing the release rate of caffeine in pH 7 buffer solution [39].

The release profiles for caffeine from HEMA/AA/AAm and HEMA/AA/MBA also have significant differences when they are compared to each other. The release of caffeine is slower in the HEMA/AA/AAm copolymer for both release media, as observed in Figure 7a,b. Caffeine is completely released after 130 min for HEMA/AA/MBA and after 210 min for HEMA/AA/AAm at pH 7. Additionally, 50% of the caffeine release is reached at 170 min for HEMA/AA/MBA and at 240 min for HEMA/AA/AAm at pH 4. These observations suggest that there are stronger interactions between the caffeine and the HEMA/AA/AAm matrix when compared to the HEMA/AA/MBA network. It is hypothesized that this is because the primary amide group in AAm is a better hydrogen bond donor than the secondary amide groups in MBA [40]. Thus, the amide groups in caffeine molecules could create stronger bonds with AAm than those with MBA, which results in a slower release of caffeine from the HEMA/AA/AAm hydrogel.

Figure 8a shows the caffeine release for the BMA/AA/AAm hydrogel at pH 4 and pH 7. The release rate is very fast in both buffer solutions, and caffeine is completely released after 45 to 55 min. Both release curves are very close to each other. This can be attributed to the structure of the hydrogel. The presence of the hydrophobic BMA segments has the effect of inhibiting the pH response character of the hydrogel. It was previously shown in Figure 4b that the swelling capacity of the BMA/AA/AAm copolymer is almost the same in the pH range between 4 and 7. This behavior suggests that the structure of the hydrogel network is nearly the same at pH 4 and pH 7. Moreover, the nonpolar BMA segments inhibit the interactions of caffeine with the copolymer, resulting in a rapid diffusion of caffeine molecules through the matrix. Finally, Figure 8b shows the release profile of caffeine from the BMA/AA/MBA hydrogel at pH 4 and pH 7. It can be observed that the release process is pH-sensitive. This suggests that the release of caffeine in the chemically crosslinked delivery system is influenced by the swelling process. The relaxation of the polymer chains in the BMA/AA/MBA hydrogel changes as a function of pH, affecting the rate of diffusion of caffeine.

### 3.5. Drug Transport Mechanism

The primary mechanism of release for many drugs from hydrogels is diffusion, which occurs through the space available between macromolecular chains. The drug release profile for a complex structure of a polymer network such as a swelling polymer depends on the relative rate of diffusion of water into the polymer matrix and the swelling of the polymer. The drug release profiles have been classified into three diffusion mechanisms [41]: Case I or Simple Fickian Diffusion, Case II Diffusion, and Case III or Non-Fickian Diffusion. When drug diffusion through the hydrogel occurs at a much slower rate than the relaxation of the polymer chains, the transport mechanism is diffusion-controlled (Case I). On the contrary, when the rate of relaxation of the polymer chains is slow relative to the rate of diffusion, the transport mechanism is relaxation-controlled (Case II). The anomalous region between Case I and Case II defines the Non-Fickian Diffusion (Case III). According to Ritger and Peppas [42], the following power law expression can be used to describe the drug release from swellable hydrogels:

(4) $$\frac{M_{t}}{M_{\infty }}=kt^{n}$$

where $M_{t}$ is the amount of drug released at time t, $M_{\infty }$ is the amount of drug released at equilibrium, *k* is a proportionality constant, which is characteristic of the drug-polymer system, and *n* is defined as the diffusional exponent of the drug release. This mathematical model is valid only for the first 60% of the drug release [43].

The values of the parameters of the Ritger–Peppas model, diffusional exponent *n*, and constant *k* have been evaluated for the release of caffeine drug from the starch-based graft copolymers, and the results are presented in Table 2 and Table 3.

Simple Fickian diffusion is characterized by a value of the diffusional exponent *n* less or equal to 0.5, while a relaxation-controlled mechanism (Case II) is characterized by a value of *n* greater or equal than 1. A value of the diffusional exponent between 0.5 and 1.0 defines an anomalous non-Fickian mechanism of diffusion, where both the diffusion and polymer relaxation mechanism are relevant to the overall rate of the drug release [44].

The use of the Ritger–Peppas model allows us to distinguish the different transport mechanisms that dominate the caffeine release process of our starch-based copolymer hydrogels. In formulations containing AAm, the release rate of caffeine is dominated by a simple Fickian diffusion at both pH = 7 and pH = 4. This suggests that the interactions between caffeine and the monomers determine how fast the caffeine is released. As a response to pH stimuli, the dry hydrogel swells due to electrostatic repulsion and increases the volume of the network. Consequently, the pore size of the porous hydrogels increases, allowing the drug to be released. The rate of the drug release is inversely proportional to the resistance to flow. The Fickian diffusion-controlled process occurs when the effective diffusive resistance is greater than the resistance of the hydrogel to undergoing a change in shape and increase in volume.

On the contrary, in formulations containing MBA, the dominant mechanism of transport depends on the pH of the release media. They exhibit a relaxation-controlled mechanism of diffusion (Case II) at pH 4, and an anomalous non-Fickian mechanism at pH 7. This behavior can be explained by the pH-sensitive character of the hydrogels containing MBA. The release rate of caffeine can be faster or slower depending on whether the hydrogel is swelling or shrinking, respectively (i.e., the relaxation forces determine the rate of diffusion of caffeine in the polymer matrix).

## 4. Conclusions

In this study, pH-responsive starch-based hydrogels were prepared successfully by the graft copolymerization of AA and other acrylic monomers (HEMA, AAm, BMA). The hydrophobic/hydrophilic character of the monomers has a crucial influence on the swelling properties of the hydrogels. It was observed that the release rate of caffeine could be controlled by the composition of the copolymer. A slow release was obtained with the AA/HEMA/AAm copolymer, and a fast release was exhibited by the AA/BMA/AAm hydrogel. The transport mechanism of caffeine release at pH 4 was controlled by Fickian diffusion in formulations containing AAm and by the polymer relaxation mechanism in formulations containing MBA. The starch-based hydrogels studied in this work show a promising application as oral drug carriers, especially for intestinal-targeted drug delivery.

## Figures and Tables

**Table 1:** Summary of copolymerization experiments: amount of comonomers and crosslinker.

Experiment	Copolymer	Starch	AA	HEMA	BMA	AAm	MBA
1	HEMA/AA/AAm	2 g	1.5 g	1.5 g	-	0.05 g	-
2	HEMA/AA/MBA	2 g	1.5 g	1.5 g	-	-	0.05 g
3	BMA/AA/AAm	2 g	1.5 g	-	1.5 g	0.05 g	-
4	BMA/AA/MBA	2 g	1.5 g	-	1.5 g	-	0.05 g

**Table 2:** Estimated parameters obtained from fitting the caffeine release experimental data at pH 4 to the Ritger–Peppas model.

Hydrogel	*n*	*k*
HEMA/AA/AAm	0.61	0.0235
HEMA/AA/MBA	1.23	0.0005
BMA/AA/AAm	0.27	0.2700
BMA/AA/MBA	1.67	0.0018

**Table 3:** Estimated parameters obtained from fitting the caffeine release experimental data at pH 7 to the Ritger–Peppas model.

Hydrogel	*n*	*k*
HEMA/AA/AAm	0.19	0.2790
HEMA/AA/MBA	0.86	0.0499
BMA/AA/AAm	0.17	0.4506
BMA/AA/MBA	0.84	0.1909
